# Retrospective Analysis of Archived Pyrazinamide Resistant *Mycobacterium tuberculosis* Complex Isolates from Uganda—Evidence of Interspecies Transmission

**DOI:** 10.3390/microorganisms7080221

**Published:** 2019-07-29

**Authors:** Sylvia I. Wanzala, Jesca Nakavuma, Dominic Travis, Praiscillia Kia, Sam Ogwang, Wade Ray Waters, Tyler Thacker, Timothy Johnson, Syeda Anum Hadi, Srinand Sreevatsan

**Affiliations:** 1 College of Veterinary Medicine, Animal Resources and Biosecurity, Makerere University, Kampala 0414, Uganda; 2 Department of Veterinary Population Medicine, College of Veterinary Medicine, University of Minnesota, St. Paul, MN 55108, USA; 3 Joint Clinical Research Center (JCRC), Lubowa 0414, Uganda; 4 National Animal Disease Center, USDA, Ames, IA 50010, USA; 5 Department of Veterinary Biomedical Sciences, College of Veterinary Medicine, University of Minnesota, St. Paul, MN 55108, USA; 6 Department of Pathobiology and Diagnostic Investigation, College of Veterinary Medicine, Michigan State University, East Lansing, MI 48824, USA

**Keywords:** tuberculosis, interspecies, transmission, whole genome sequencing, SNPs, zoonosis, bovine TB, pyrazinamide, isoniazid

## Abstract

The contribution of *Mycobacterium bovis* to the proportion of tuberculosis cases in humans is unknown. A retrospective study was undertaken on archived *Mycobacterium tuberculosis* complex (MTBC) isolates from a reference laboratory in Uganda to identify the prevalence of human *M. bovis* infection. A total of 5676 isolates maintained in this repository were queried and 136 isolates were identified as pyrazinamide resistant, a hallmark phenotype of *M. bovis*. Of these, 1.5% (*n* = 2) isolates were confirmed as *M. bovis* by using regions of difference PCR analysis. The overall size of whole genome sequences (WGSs) of these two *M. bovis* isolates were ~4.272 Mb (*M. bovis* Bz_31150 isolated from a captive chimpanzee) and 4.17 Mb (*M. bovis* B2_7505 from a human patient), respectively. Alignment of these genomes against 15 MTBC genome sequences revealed 7248 single nucleotide polumorphisms (SNPs). Theses SNPs were used for phylogenetic analysis that indicated a strong relationship between *M. bovis* and the chimpanzee isolate (Bz_31150) while the other *M. bovis* genome from the human patient (B2_7505) analyzed did not cluster with any *M. bovis* or *M. tuberculosis* strains. WGS analysis also revealed multidrug resistance genotypes; these genomes revealed *pncA* mutations at positions H57D in Bz_31150 and B2_7505. Phenotypically, B2_7505 was an extensively drug-resistant strain and this was confirmed by the presence of mutations in the major resistance-associated proteins for all anti-tuberculosis (TB) drugs, including isoniazid (*Kat*G (S315T) and *Inh*A (S94A)), fluoroquinolones (S95T), streptomycin (rrs (R309C)), and rifampin (D435Y, a rare but disputed mutation in *rpoB*). The presence of these mutations exclusively in the human *M. bovis* isolate suggested that these occurred after transmission from cattle. Genome analysis in this study identified *M. bovis* in humans and great apes, suggesting possible transmission from domesticated ruminants in the area due to a dynamic and changing interface, which has created opportunity for exposure and transmission.

## 1. Introduction 

*Mycobacterium bovis*, a subspecies of the *Mycobacterium tuberculosis* complex (MTC), is a significant cause of bovine tuberculosis (bTB) in the developing world [1,2]. Each MTC member displays varied pathogenicity, geographical distribution, epidemiology, and host range [3]. MTCs, the causative agents of tuberculosis, are intracellular pathogens that have very low (0.01–0.03) synonymous nucleotide variation with no significant trace of genetic exchange among them [4]. In the developed world, bovine TB (bTB) has nearly been eradicated from domesticated animals, thus the risk of human transmission is low. This was achieved through mandatory testing of cattle, removal of positive reactors and in-contact animals, and compulsory pasteurization of milk [5,6,7]. In contrast, in resource-limited countries where the devastation of MTCs on humanity continues unabated, effective disease-control measures, like mandatory milk pasteurization and removal of positive reactors, is difficult to implement mainly due to economic factors [8,9,10,11,12]. For example, bTB is not reportable in many countries in sub-Saharan Africa and there are no compensatory mechanisms in place for farmers whose animals test positive for the disease [6,13,14,15].

The World Health Organization (WHO) estimates that two billion humans are infected with latent tuberculosis, with a 5% to 10% chance of developing active TB [16,17]. The brunt of this burden is largely borne by developing countries, with an approximate two million deaths annually, 95% of which are in low or medium-income countries [16]. The proportion of tuberculosis infections caused by *M. bovis* is not well-defined especially in resource-limited settings of the world. Prevalence estimates of *M. bovis* range from 5% to 16% [13,15]. In Uganda, routine TB testing does not assign species within MTC organisms, hence any human infection with an MTC is assumed to be due to *Mycobacterium tuberculosis (sensu stricto)*. In 2015, Uganda had an estimated population of 39 million people, with about five laboratories that carry out routine TB screening with culture and drug sensitivity testing; these are all located in urban centers [18,19]. Staining for acid-fast bacilli (AFB) on sputum smears to screen for TB is carried out by a larger number of network laboratories. Animal TB diagnostics in Uganda are lagging far behind human TB diagnostics and available bTB information is based on passive surveillance data from abattoirs that are active in recording and submitting annual cattle slaughter reports [20]. Uganda does not have a national bovine TB eradication program and previous studies on bTB in Uganda have shown the presence of *M. bovis* among cattle belonging to the pastoral communities [20,21,22]. 

Even though *M. tuberculosis* is the most prevalent of the *M. tuberculosis* complex in Kampala, infections with *M. tuberculosis* or *M. bovis* are clinically and pathologically indistinguishable [13,23]. The challenge is physicians treat both diseases similarly although *M. bovis* is naturally resistant to pyrazinamide, one of the first-line TB drugs [24]. More importantly, species assignment of MTCs provides a possible eco-source of the agent that can aid in appropriate treatment and prevention. It is therefore imperative that MTC isolates are completely identified to the subspecies level in order to provide sufficient information on their epidemiology and enable appropriate treatment of the patients [24].

There is little documented information about the proportion of people infected by *M. bovis* in Uganda. This work was developed to determine and differentiate archived human MTC isolates, from a large reference laboratory, Joint Clinical Research Centre (JCRC), Kampala, Uganda, as *M. bovis* or *M. tuberculosis* (MTB). The JCRC is an HIV/AIDS care and research institution established in 1990 to provide a scientific approach to the national HIV/AIDS problem [25]. This retrospective study characterized archived pyrazinamide resistant isolates stored at the JCRC from 1999 through 2012.

## 2. Materials and Methods

### 2.1. Institutional Review Board (IRB) and Ethical Approvals 

Permission to use isolates for molecular characterization was obtained from the research committee of the JCRC; the JCRC Uganda-Case Western Reserve University (CWRU) Research Collaboration and the Molecular Diagnostics Laboratory Department of Medical Microbiology, College of Health Sciences (CHS), Makerere University Kampala. Institutional Review Board (IRB) approval was obtained from the Makerere University, School of Biomedical Sciences Research and Ethics Committee. Scientific and ethical clearance was obtained from the Uganda National Council for Science and Technology (UNCST) reference number HS 1478. Since the work was a retrospective study using archived isolates, this work was exempt from University of Minnesota IRB approval. 

### 2.2. Study Site 

The archived MTC isolates were obtained from the JCRC in Kampala, Uganda [25]. Confirmatory tests to identify *M. bovis* were done at the Molecular Biology Lab in the Department of Microbiology, College of Health Sciences, Makerere University. 

### 2.3. Study Samples 

Pyrazinamide (PZA) resistant isolates were drawn from a collection of 5676 archived isolates collected between 1999 and 2012 from 19 different research studies previously conducted in partnership with key research collaborators at JCRC (Figures S1 and S2).

We first determined the proportion of *M. tuberculosis* isolates resistant to first-line anti-tuberculosis drugs (pyrazinamide, rifampin, isoniazid, and ethambutol and streptomycin), and then carried out comparative genomics. Furthermore, we sought to determine if there was a non-random distribution of genomotypes of MTC organisms infecting animals and humans in Uganda using a defined single nucleotide polymorphism (SNP) set to differentiate the *Mycobacterium tuberculosis* complex group of organisms and enable determination of drug resistance and characterization of these MTC isolates as drug resistant (DR), multi-drug resistant (MDR), or extremely drug resistant (XDR) organisms.

### 2.4. Sampling, Culture, and Isolation of Mycobacteria 

All the work with the MTC isolates was carried out in biosafety level three (BSL-3) containment facilities. Archived MTC isolates from the JCRC were retrieved from −80 °C freezer stocks, thawed and inoculated into Middlebrook 7H9^®^ broth, and incubated at 37 °C for 42 days [26,27]. The samples were sub-cultured on solid media, Middlebrook 7H10^®^ (Beckton and Dickinson, Heidelberg, Germany) containing sodium pyruvate, and Oleic Acid-albumin-Dextrose-Catalase (OADC^®^) enrichment. In case of contaminating growth, the cultures were also sub-cultured on selective media, Middlebrook 7H11^®^ containing sodium pyruvate, glucose, sterile calf serum, OADC enrichment, sheep blood, malachite green, and glycerin, and incubated at 37 °C. The cultures were inspected weekly and considered negative when there was no visible growth after 8 weeks. Ziehl Neelsen and auramine fluorescent staining and microscopy were carried out on the isolates to confirm that they were *Mycobacterium* sp.

### 2.5. Molecular Testing to Confirm MTC 

Isolates were harvested and inoculated in Middlebrook 7H9^®^ broth in cryovials and taken to the Molecular Diagnostic Laboratory, Department of Medical Microbiology, Makerere University for heat killing, DNA extraction, and PCR-based regions of difference (RD) analysis to differentiate *M. bovis* from other members of MTC. DNA was extracted by sonication and quantified using the GeneQuant^®^ (Biochrom, Holliston, MA, USA) UV spectrophotometer (20–30 μg per isolate) (26). Genomic regions of difference (RD analysis) for RD1, RD9, RD4, and RD12 were used to differentiate among MTC species as described [24,28,29,30]. These RDs are regions within the MTC genome that represent a loss of genetic material in *M. bovis* compared to *M. tuberculosis* H37Rv [24,31,32]. 

### 2.6. DNA Extraction/DNA Preparation for PCR 

Cells stored in 7H9 at −20 °C were harvested and heat killed at 95 °C for 1 h in a hybridization oven and DNA was extracted by sonication. Heat killed samples were centrifuged at 15,700 rcf or 13,000 rpm (Eppendorf 5415D Centrifuge; Eppendorf, Hauppauge, NY, USA) for 2 min in a microfuge, supernatant discarded, and pellet retained. To the pellet, 500 µL of Tris-EDTA buffer, pH 8, was added and centrifuged at 13,000 rpm for another 2 min and the pellet was retained after pouring off the supernatant. The pellet was re-suspended in 100 µL Tris-EDTA buffer and sonicated in an ultra-sonic bath (Bandelin Sonorex Ultrasonic BathRK100H (Schalltec, Moerfelden, Germany)) for 45 min. The resultant solution was centrifuged at 12,000 rpm for 10 min to obtain the supernatant that contained DNA. These were temporarily stored at −20 °C prior to PCR analysis [26].

### 2.7. Genomic Deletions (Regions of Difference) Analysis

The target gene loci, primer names, primer sequences, amplification product sizes, and their genome locations in *M. tuberculosis* H37Rv are listed in Table 1. IS*6110* PCR was conducted to confirm that the samples belonged to the *Mycobacterium tuberculosis* complex [30,33]. IS*6110* is an insertion element present in the DR region of most *M. tuberculosis* strains [32]. Briefly, a master mix consisting of PCR water (Qiagen^®^, Germantown, MD, USA), 2xTaq master mix (Qiagen^®^), forward and reverse primers P_43_ (5′-TCA GCC GCG TCC ACG CCG CCA-3′) and P_53_ (5′-CCG ACC GCT CCG ACC GAC GGT-3′) was used. The PCR program entailed an initial denaturation at 95 °C for 5 min, 28 cycles of denaturation at 95 °C for 1 min; annealing at 55 °C for 30 s; extension at 72 °C for 30 s, and final extension at 72 °C for 10 min. The amplicons were electrophoresed on a 1.5% agarose gel stained with ethidium bromide and visualized on UV bio-imager^TM^ (Ontario, Canada). *Mycobacterium tuberculosis* strains H37Rv and *M. bovis* JN55 [34] were positive controls and nuclease free water was the negative control. Confirmed MTC positive samples were analyzed for RD9, RD12, RD4, and RD1 to identify any *M. bovis* amongst the MTC species. Master Mix consisted of PCR water (Qiagen^®^), 2xTaq master mix (Qiagen^®^), and forward and reverse primers specific for respective regions of difference (Table 1). RD-PCR was performed using same conditions as for IS6110. Amplicons were resolved similarly on a 1.5% agarose gel and the sizes of the amplicons were estimated by comparison against a 100 bp DNA ladder. *M. tuberculosis* strains H37Rv and *M. bovis* JN55 were consistently used as the positive controls and nuclease free water as the negative control. A negative PCR result for RD9, RD12, and RD4 and a positive reaction for RD1 confirmed the presence of *M. bovis* [24].

### 2.8. Genomic Analysis—Sequencing, Assembly, and Annotation of Eight Genomes

Ten pyrazinamide resistant isolates were selected and shipped to the Broad Institute, USA for whole genome sequencing, WGS [35]. Eight of the 10 mycobacterial DNA extracts were subjected to whole genome shotgun sequencing from a paired-end library at the Broad Institute using the Illumina HiSeq 2000 platform (GA Pipeline version RTA1.17.21.3; Illumina, Palo Alto, CA, USA) [36]. Eight draft genome sequences together with 9 other genomes from different regions of the world and reference genomes *Mycobacterium bovis* Af2122/97 (GenBank database-accession no. BX248333) and *M. tuberculosis* CDC 1551 were downloaded from NCBI at ftp://ftp.ncbi.nih.gov/genomes/ in FastA format for genome alignment with Progressive Mauve [37,38] and then against AF2122/97 as a reference genome. Close neighbor references from the *Mycobacterium tuberculosis* complex were used to identify orthology. The Progressive Mauve software was used to align homologous regions of the genomes in order to identify evolutionary changes in their DNA [38]. This software constructs multiple genome alignments in the presence of large-scale evolutionary events, like rearrangement and inversions, and uses these multiple genome alignments to provide a basis for comparative genomics and the study of genome-wide evolutionary dynamics. Single nucleotide polymorphisms (SNPs) were extracted from CLC workbench genome alignments and used to infer phylogeny with phylogenetic tree construction using Mega 7.0 (Molecular Evolutionary Genetic Analysis software; https://www.megasoftware.net) using the maximum likelihood method with 1000 bootstrap replicates under the Hasegawa–Kishino–Yano (HYK) model [39]. These were aligned against the reference strain (*M. bovis* AF2122/97) to determine phylogenetic relatedness. 

All the genome sequences were re-annotated with RAST (Rapid Annotation using Subsystem Technology), which is a fully automated server for complete or draft archaeal and bacterial genomes [40]. Comparisons were done using BLAST (Basic Local Alignment Search Tool) at https://blast.ncbi.nlm.nih.gov/Blast.cgi and the results are displayed as graphical maps showing sequence features, gene and protein names, COG category assignments, and sequence composition characteristics [40,41]. Further, the presence of the following antibiotic resistance genes, *pnc*A, *kat*G, *rpo*B, *inh*A, *emb*ABC, *gyr*A, and 16S rRNA (*rrs*), from the reference strain *M. tuberculosis* H37Rv were determined using the NCBI tBLASTx tool (translated nucleotide using a translated nucleotide query), where each gene served as a query sequence for alignment against each of the different genomes in this study. This was to determine the exact location of the drug-resistance associated mutations in the different genomes. All regions associated with drug resistance were re-sequenced using the Sanger sequencing method to confirm polymorphisms in these targets.

## 3. Results

### 3.1. Culture Results 

Out of 5676 stored MTC isolates, only 136 (2.4%) were resistant to PZA, a first line anti-TB drug. These 136 isolates were retrieved and verified as MTC based on typical growth on media with and without antibiotics, positive results with Ziehl Neilsen and auramine staining and microscopy, and no growth on blood agar. Selective amplification of the *Mycobacterial* IS*6110* gene, an MTC specific insertion sequence, confirmed the presence of MTC in 133 of 136 DNA samples. Three of the samples gave indeterminate results and hence were removed from further processing steps. The sample sources are indicated in Figure S3 of the Supplementary Figures.

### 3.2. Regions of Difference (RD) Analysis: RD9, RD4, RD12, and RD1 

**Figure 1:**
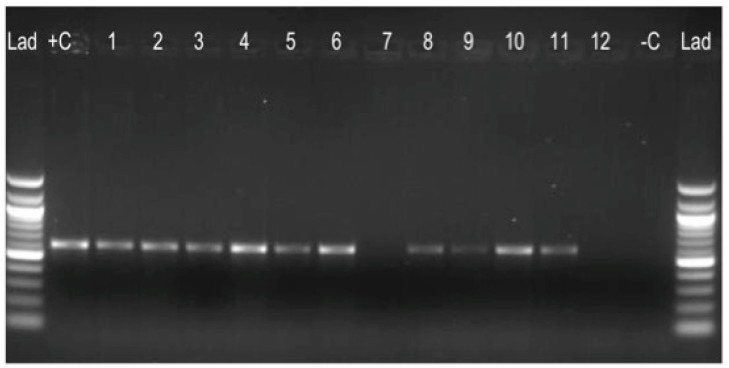
PCR result for RD9 (600 bp) for some of the 133 IS*6110* positive samples. Order on gel: 100 bp Lad, +C: Positive control (*Mycobacterium tuberculosis* strain H37Rv), samples 1–12, -C; negative control (nuclease free PCR water).

Of the 133 IS*6110* positive samples subjected to RD analyses, 126 were RD9 positive while 7 (5.3%) samples were negative. The RD9 positive results suggested that the isolates were either *M. tuberculosis*, *M. canetti*, or *M. africanum* subtype II. These were not analyzed further. The RD9 negative isolates were further analyzed with RD4 to further delineate the MTC species therein. Of the seven RD9 negative samples analyzed with RD4, five were RD4 positive and two RD4 negative. RD4 is a region deleted from all *M. bovis* strains (that is, classical *M. bovis*, *M. bovis* BCG, and MOTTs) but is present in *M. canettii, M. tuberculosis, M. africanum* type I and II, and *M. microti* isolates. To confirm the presence of either classical *M. bovis* or *M. bovis* BCG, the RD4 negative samples were analyzed further using both RD12 and RD1 PCR. The two RD4 negative samples were analyzed for the presence of RD12 and RD1 loci. Both isolates were RD12 negative, confirming them as *M. bovis.* Two isolates were positive for RD1. A summary of the RD analyses is presented in Table 2. These samples were further characterized by genome sequencing (Figure 1).

The prevalence of *M. bovis* in archived PZA samples at JCRC was determined to be at 1.5% (2/136). The two *M. bovis* positive samples were Bz_31150 and B2_7505. Sample Bz_31150 was a bronchial wash from a chimpanzee submitted for characterization to the JCRC in May 2007. This sample was sensitive to streptomycin, isoniazid, rifampicin, and ethambutol, first-line anti-tuberculosis drugs. It was resistant to pyrazinamide, which is expected for *M. bovis* since this organism carries inherent resistance to the drug [13,24,30]. Another sample taken for sequencing was B2_7505, a sputum sample that was a classical *M. bovis* isolate, from a human patient collected in 2006. This person was part of a multi-resistant TB drug study. This isolate was resistant to all the anti-tuberculosis antibiotics. 

### 3.3. Phenotypic Drug Susceptibility Profiles 

All 136 isolates were resistant to at least one anti-TB drug (Figure S4). This was very typical of the trend of drug resistance among the 5676 isolates scanned for analysis in this study. In Figure S4 and Table 3, less than 30% of the isolates were sensitive to the first-line TB drugs and 83 (61%) out of 136 of the isolates were resistant to all first-line TB drugs (e.g., isoniazid, rifampicin, pyrazinamide, and ethambutol) and streptomycin. These patients were characterized as having multi-drug resistant TB or MDR-TB. Twenty-six or 19% of the isolates were sensitive to all first-line TB drugs except for PZA. In addition, 44% or 32% of the isolates were resistant to all the four first-line TB drugs and streptomycin. 

### 3.4. Mycobacterium Bovis from Human Host Among Sequenced Genomes

Eight genomes sequenced by Illumina were uploaded at NCBI [42,43] and the Olive database [36] (Table 4). Two of these isolates were *M. bovis* and six were *M. tuberculosis.* Illumina HiSeq2000 whole genome sequencing was performed, with 1.1 M spots yielding 214.9 Mb for *M. bovis* BZ_31150, and 1.8 M spots yielding 356.7 Mb for *M. bovis* B2_7505. For *M. bovis* Bz_31150, Illumina reads were assembled into 141 contigs and 136 scaffolds, resulting in a genome size of ~4.272 Mb, with a GC content of 65.54% at 24x coverage [44]. For *M. bovis* B2_7505, Illumina reads were assembled into 243 contigs and 206 scaffolds, resulting in a genome size of ~4.17 Mb, with a G + C content of 65.5% at 31.0× coverage [44]. All other genomes sequenced in this study provided at least 30-fold coverage, but the genomes could not be closed due to repetitive region redundancy. Nevertheless, the genome coverage was excellent and provided confidence to extract SNPs from regions that aligned with AF2122/97 at >20-fold coverage.

### 3.5. SNPs Obtained by Phylogenetic Analysis

**Figure 2:**
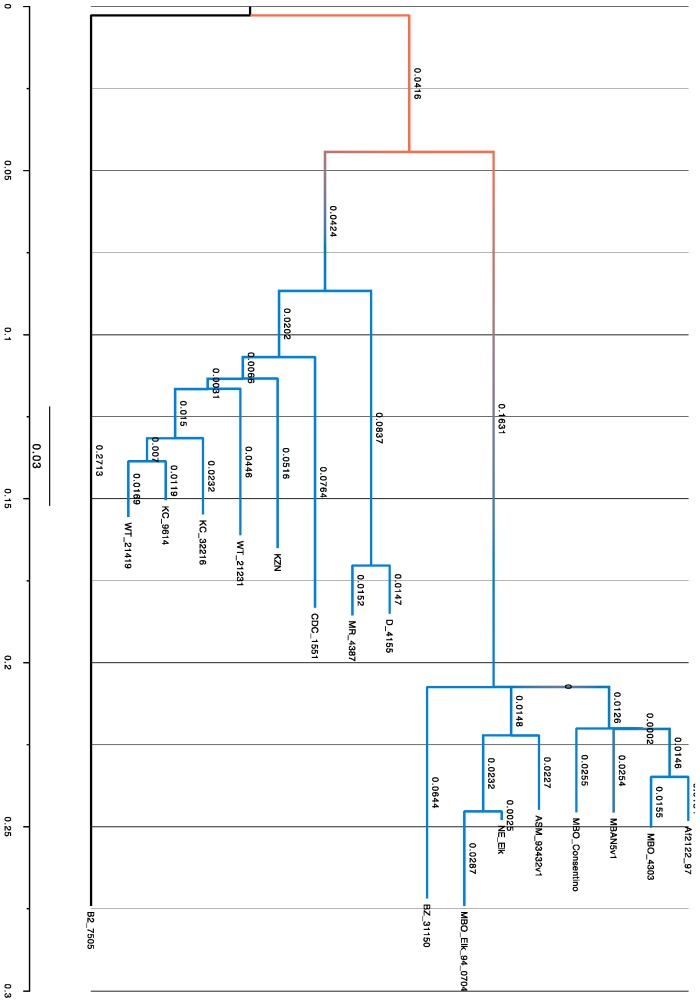
Molecular phylogenetic analysis of *Mycobacterium tuberculosis* complex organisms by the maximum likelihood method. Evolutionary history was inferred by using the maximum likelihood method based on the Hasegawa–Kishino–Yano model (94). The tree with the highest log likelihood (-49,783.9307) is shown. The percentage of trees in which the associated taxa clustered together is shown next to the branches. Initial tree(s) for the heuristic search were obtained automatically by applying Neighbor-Join and Bio neighbor joining algorithms to a matrix of pairwise distances estimated using the maximum composite likelihood (MCL) approach, and then selecting the topology with the superior log likelihood value. The tree is drawn to scale, with branch lengths measured in the number of substitutions per site. The analysis involved 17 nucleotide sequences. Codon positions included were first + second + third + noncoding. All positions containing gaps and missing data were eliminated. There was a total of 7248 positions in the final dataset.

In total, 7248 SNPs were extracted across the 17 genomes (Table 5) and used to create a phylogenetic tree in MEGA 7.0 using the maximum likelihood method with 1000 bootstrap replicates and the Hasegawa–Kishino–Yano (HYK) model (Figure 2). Phylogenetic analysis of the SNP distribution on genome sequences was carried out to determine the relationships between the isolates and reference genomes as well as likely disease transmission patterns. In our analysis, *M. bovis* from a human being (B2_7505) was distinct as it neither clustered with any *M. bovis* nor *M. tuberculosis* strains (Figure 2).

In addition, we carried out genome annotation of all the eight Ugandan genomes and comparative analysis of the two *M. bovis* genomes (Bz_31150 and B2_7505) with the reference genome *M. bovis* Af2122/97, *M. bovis* BCG, and *M. tuberculosis* CDC_1551 with the RAST tool [40] 

### 3.6. Genome Analysis of Multi-Drug Resistance Mutations

Mycobacteria lack diversity and this implies that when amino acid polymorphisms are detected, it strongly suggests that the variation has functional importance, like antibiotic resistance [45] (Table 6 and Table 7). All the Ugandan isolates were phenotypically resistant to PZA, but on genome analysis, they had mutations in different locations of the 561 bp *pnc*A gene. The animal isolates (*M. bovis* Af2122/97, *M. bovis* Bz_31150, *M. bovis* B2_7505, *M. bovis* Elk strain, and *M. bovis* Corsentino strain) and the human isolate *M. bovis* B2_7505 had the same *pnc*A single C→G point mutation at nucleotide 169, which resulted in an H57D substitution [46]. In addition, both B2_7505, the human TB isolate, and Bz_31150, the chimpanzee isolate, had an adenine residue at nucleotide 285 in the *oxy*R gene, further confirming them as *M. bovis* isolates [47].

Two Ugandan MTb strains (*M. tuberculosis* D_4155 and *M. tuberculosis* KC_32216) were phenotypically PZA resistant but exhibited no mutation at the genome level while *M. tuberculosis* KC_9614 had a mutation four nucleotides upstream of the *pnc*A gene Y(-4)C. This agrees with Sreevatsan et al.’s, [46] work, where 28% of the PZA resistant *M. tuberculosis* organisms they tested lacked nucleotide sequence changes in *pnc*A. The remaining PZA resistant *M. tuberculosis* organisms displayed unique single point mutations; WT_21419 had a V163A mutation, MR_4387 had an A134V mutation while WT_21231 had a *pnc*A V131F mutation. AhpC is an alkyl hydroperoxide reductase C, an enzyme that reduces organic peroxides, and it has been shown to be involved in the response to oxidative stress in mycobacterial species. The *ahp*C gene is located adjacent to the *oxy*R gene and there were mutations identified in the *ahp*C gene in two isolates; in MR_4387, there were G(-88)A and T(-76)A nucleotide mutations while in D_4155, there was a G(-88)A mutation upstream of *ahp*C. 

More than 95% of rifampicin (RMP) resistance is associated with mutations in an 81 bp region of the *rpo*B gene (the RIF resistance determining region (RRDR) from codons 507 to 533 of *rpo*B), but isoniazid (INH) resistance is more complex and has been associated with multiple genes, most frequently with *kat*G and *inh*A [48]. Other common mutation patterns are found at codon 315 (55%–90%) of *kat*G and codon 43 (47%–79%) of *rps*L for streptomycin resistance [49]. Mutations in the *kat*G and *inh*A genes together may be responsible for 80% of all organisms resistant to INH [46]. In our work, *M. tuberculosis* KC_9614 had the S315T mutation in the *kat*G gene (catalase-peroxidase) while *M. bovis* B2_7505 had both the S315T mutation in *kat*G as well as the S94A mutation in the *inh*A gene (NADH-dependent enoyl-reductase), defining it as an INH resistant organism [50].

Phenotypically, *M. bovis* B2_7505 and *M. tuberculosis* isolates MR_4387, KC_9614, WT_21419, and WT_21231 were all resistant to rifampicin (RMP) yet the mutations they exhibited at the genome level fell outside the RRDR (e.g., S450L, D435Y), which is meant to convey high-level resistance [51]. Gagneux et al. posit that rifampicin resistance-conferring mutations associated with no or low fitness cost in vitro are the most frequent in clinical strains [51]. Several isolates, including the reference strain, *M. bovis* Af2122/97, had the R463L mutation in *kat*G, but according to Sekiguchi et al., the R463L mutation is not known to be associated with INH resistance [50,52]. This means that the phenotypic resistance to INH observed in some of the isolates (MR_4387, WT_21419, and WT_21231) was probably due to mutation elsewhere. Furthermore, there was an *rpo*B S531L mutation in WT_21231 and WT_21419 and a D516V mutation for MR_4387, which are classic mutations associated with high-level RMP resistance [53].

Mutations in the *gyr*A gene related to fluoroquinolone (FQ) resistance were seen in several isolates, with the most common being E21Q, G668D, and T80A; all these mutations were outside the QRDR yet these isolates demonstrated phenotypic resistance [54]. Isolates B2_7505, Bz_31150, D_4155, and MR_4387 had a C206G nucleotide mutation in *gyr*A. In addition, *M. bovis* B2_7505 also had a S95T mutation in the QRDR. Fluoroquinolone resistance has been associated with alterations in DNA gyrase, especially mutations in the quinolone resistance determining region (QRDR) of *gyr*A (codons 74 to 113) and *gyr*B (codons 500 to 538), respectively [54,55]. Its estimated that 60% to 90% of Mtb clinical isolates with extremely high FQ resistance have mutations in the QRDR of gyrA, particularly codons 88, 90, 91, and 94 [54,56]. 

For ethambutol resistance, a G884D mutation was identified in *emb*A of MR_4387 and D_4155, Mtb isolates. In addition, there was a T608N mutation in the *emb*A in KC_9614. None of these are documented mutations in the literature for EMB resistance. The mutations for *emb*C were only in the *M. bovis* isolates except for B2_7505 (see Table 6 and Table 7). 

## 4. Discussion 

Although much has been studied on tuberculosis in developed parts of the world and Asia, there are still gaps in the knowledge on tuberculosis—particularly zoonotic tuberculosis—in Africa. In this study, we carried out comparative genomics on archived pyrazinamide resistant MTC isolates from a multicenter study archive in Uganda to determine if there was a non-random distribution of genomotypes of MTC organisms infecting humans and animals in Uganda with the use of genome-wide single nucleotide polymorphisms (SNPs) to differentiate MTC organisms. The guiding hypothesis was whether there was multi-directional transmission of tuberculosis occurring in resource-limited settings, where animal-human contact is intimate and frequent.

Cattle are the main hosts for bTB in Uganda and the region, though there are studies that indicate that goats can be a source of infection as well, but TB in small ruminants is rarely detected at the abattoir as a result of a lower quality of meat inspection than that performed for cattle [8]. In Uganda, milk is usually boiled, but raw and fermented milk are also consumed by some pastoral communities [20,21,22,57]. With the absence of an active bTB surveillance program in Uganda, there is still little known about the scale of the problem in both the animal and the human population. Pasteurized milk is unaffordable for many people, and this coupled with the consumption of raw milk among the pastoral communities, raises important questions about the actual bovine TB status of the country. 

Bovine TB generally results in extra-pulmonary lesions. Thus, a bias toward detection on a very small proportion of *M. bovis* was anticipated as about 80% of the PZA resistant isolates came from sputum. There are limited laboratory facilities for the culture and typing of tubercle bacilli in Uganda especially in rural areas [11,58]. Sputum smear microscopy with acid-fast stains is the primary approach to pulmonary TB detection in Ugandan TB laboratories. It is an easy, fast, and inexpensive way to screen for TB cases but has low sensitivity, especially with persons co-infected with HIV. Furthermore, *M. bovis* infections are generally missed when microscopy on sputum alone is used for diagnostics and as a triage for culturing for mycobacteria.

The World Health Organization indicates that bovine TB is a neglected disease [16] and given its insidious nature, policy makers need to address it. There is still little published information about the relative proportion of MTC due to *M. bovis*, the epidemiology as well as the public health aspects of this disease [11,59,60,61]. In fact, Ayele et al. [62] proposed that of all the reports submitted to the Office of International Epizootes (OIE) and WHO by different African member countries, none mention bTB as an important human TB infection. Tanzania, one of the countries neighboring Uganda in East Africa, is one of the few countries in Africa that has quantitative data on the bTB prevalence in humans, with more than 30% of recorded TB cases listed as extra-pulmonary in the Arusha region [59,63]; of these *M. bovis* was isolated from 7/65 or 10.8% culture positive cases with cervical lymphadenitis. This also highlights the critical need for physicians to be aware of *M. bovis* and the different routes of transmission and different treatment recommendations. 

### 4.1. Mycobacterium Bovis in PZA Resistant Isolates

We carried out culture, regions of difference (RD) PCR analysis as well as whole genome sequencing on archived pyrazinamide resistant isolates from Uganda and found two *M. bovis* isolates; *M. bovis* B2_7505 isolated from a human patient and *M. bovis* Bz_31150 isolated from a chimpanzee. Out of the over 5000 stored isolates at the JCRC, only 133 or 2.4% were resistant to pyrazinamide, and only two of these turned out to be *M. bovis.* We expected a higher number of isolates to be positive given that *M. bovis* is naturally resistant to PZA, a first-line anti-tuberculosis drug [13]. The rest of the PZA resistant isolates were *M. tuberculosis*, pointing to a high degree of drug resistance to anti-TB drugs amongst the humans from whom the isolates were collected (Supplementary Figure S4). In addition to resistance to PZA, the case patients were resistant to multiple anti-tuberculosis drugs (MDR-TB).

Studies in Ethiopia on bTB have indicated that a higher prevalence of *M. tuberculosis* in cattle owned by farmers with active TB than in farmers who did not have active tuberculosis; this suggests possible bi-directional transmission of mycobacterial species between cattle and their owners [64,65,66,67,68,69]. In Alemaheyu’s study [64], *M. bovis* was isolated from the sputum of cattle owners and *M. tuberculosis* from cow’s milk, which suggests bi-directional transmission of mycobacterial species. Studies in Tanzania also indicate that consumption of raw milk is an important risk factor in bTB transmission [10,13,59,70]. Humans acquire bTB infection through ingestion of infected contaminated milk and milk products and also by inhalation when there is close physical contact between the owner and their cattle, especially at night, since in some cases, they share shelters with their animals [5,64,65,71]. In this study, the person infected with *M. bovis* (sample B2_7505) was an adult female with MDR TB. Although she received treatment from the JCRC in Kampala, the capital city of Uganda, we cannot rule out the possibility that she had close contact with animals since many Ugandans do have contact with animals.

### 4.2. Whole Genome Sequencing (WGS) and Analysis

WGS provides unique diagnostic abilities for drug-resistant TB given that it provides information on entire genes and their promoters, expected sensitivity of strains, genetic background, epidemiological data, and an indication of risk of laboratory contamination giving a ‘complete genotypic profile’; features that are critical as incidences of multi-drug resistant and extensively drug resistant TB are increasing [72,73]. In this work, WGS of eight Ugandan isolates and genome analysis with Mauve [37], Mega [39], RAST [40], and the use of the PATRIC database [74] revealed that the two *M. bovis* isolates from Uganda displayed unique characteristics. The *M. bovis* reference genome Af2122/97 has a genome of ~4.3 Mb while the Ugandan isolates were ~4.272 Mb (*M. bovis* Bz_31150) and 4.17 Mb (*M. bovis* B2_7505) [43]. According to some schools of thought, evolution towards pathogenicity is often accompanied by a reduction in genome size, which is compensated in part by gene duplications and diversification [75]. It has been proposed that genome downsizing of pathogenic bacteria is as a result of adaptation to a pathogenic lifestyle that includes the exploitation of the resources of a host organism, which might make certain gene functions redundant and subject to gene loss [1,75,76]. On the other hand, the reduced genome size could be due to the fact that these were draft genomes, with some regions of the genomes not covered. Another possible reason is that it is difficult to obtain complete MTBC genome sequences with second generation sequencing technologies, like the Illumina Hiseq platform, due to the high GC content and repetitive sequences [77]. 

### 4.3. Phylogenetic Analysis

Phylogenetic analysis carried out on the Uganda isolates together with the Af2122/97 reference genome, US *M. bovis* strains isolated from Elk (*M. bovis* Elk and Corsentino strains) and isolates from Argentina and Brazil (Figure 2), show that Bz_31150 clustered with the other *M. bovis* strains. The other Uganda Mtb strains clustered with *M. tuberculosis* CDC_1551 and in particular, *M. tuberculosis* WT_21231 clustered very closely with *M. tuberculosis* KZN, the Kwazulu Natal strain isolated from a patient in South Africa. *Mycobacterium bovis* B2_7505 did not cluster with any of the two main cluster groups above but formed an intermediate group between the two. This zoonotic *Mycobacterium bovis* B2_7505 is an extensively drug resistant (XDR) strain that may have been either a mixed infection or a contaminated culture. On the other hand, this may represent a unique isolate that likely underwent multiple genomic changes in-vivo to adapt to the human host to successfully circumvent new host defenses, persisting in the host and developing resistance through various mechanisms, many of which are yet to be fully understood. This would need reconfirmation after re-culturing the bacterium and sequencing multiple individual colonies. Furthermore, long-read sequencing technologies would be very helpful in delineating mixed cultures as well as improving confidence in the genome assemblies.

Analysis of selected isolates (*M. bovis* B2_7505, *M. bovis* Bz_31150, *M. bovis* BCG, *M. bovis* AF2122/97, and *M. tuberculosis* CDC_1551) using RAST enabled us to visualize the regions of the genomes that were most similar and others that were not. This is in line with what we obtained from the phylogenetic analysis using single nucleotide polymorphisms (SNPs). 

This work demonstrates that although MTCs have a high degree of nucleotide relatedness, there are significant genomic differences between them, which can be harnessed to make critical decisions affecting tuberculosis treatment and management. SNP-based phylogenetic analysis helped to differentiate the different MTC genomes; this provides a population genetic framework to aid in identifying factors responsible for the wide host range and disease phenotypes of *M. bovis* and its behavior in different host species [78]. Our understanding of TB epidemiology and genomics is constantly being modified and improved with the rapid advances in whole genome sequencing [79,80,81,82]. 

### 4.4. Antibiotic Resistance Genes of Uganda Isolates

Resistance to drugs develops when mutations or chromosomal errors occur, in genes that encode drug targets or drug metabolic pathways, which impacts the effectiveness of the drugs. This is problematic when a patient is given a less-than-optimal treatment or there is low adherence to the drugs. In addition, lack of detection and subsequent unsuitable treatment increase the risk transmission of drug resistant tuberculosis and in fact studies have shown that a large proportion of MDR and XDR cases are due to primary transmission [48,83]. 

The *pnc*A gene encodes the pyrazinamidase (PZase) enzyme, which converts PZA, a pro-drug, into the active pyrazinoic acid [84]. PZA kills semi-dormant tubercle bacilli that are not affected by other anti-tuberculous drugs and its inclusion in the treatment of tuberculosis shortens the period of therapy from 12 to 18 months to 6 months [85,86]. In this study, we sequenced eight genomes from Uganda that were phenotypically PZA resistant but on genome analysis, three of them did not have any mutation in the *pnc*A gene (Mtb D_4155, Mtb KC_32216, and Mtb KC_9614). Further, there was no mutation in the *M. bovis* Ravenel strain we used yet this mutation (H57D) should be present in this isolate since *M. bovis* strains are naturally resistant to pyrazinamide [13,85]. This is a laboratory strain and may have mutated over the course of several passages. 

Table 6 shows all drug resistance related mutations. Table 7 shows a more detailed study of SNPs related to different TB drug resistance associated mutations. DNA sequencing studies have shown that mutations occur across the entire length of the *pnc*A gene, with some SNPs occurring more frequently than others possibly because these SNPs are rooted in ancestral strains [46,84]. This means that development of a molecular assay for PZA resistance detection would be more challenging compared to other genes, like *rpo*B, *gyr*A, and *emb*B, that have well-defined resistance-causing mutations [84]. Studies also show that mutations in the *pnc*A gene can be absent in a small proportion of isolates that are phenotypically resistant to PZA, thus it is said to be indicative of an alternative mechanism of PZA resistance other than mutation in the *pnc*A gene [84].

Isoniazid (INH) targets both active and latent tuberculosis and has a relatively low cost and toxicity, but INH resistance is quite complex and has been associated with multiple genes, usually *kat*G (S315T), which encodes a catalase peroxidase that transforms INH to its active form, and *inh*A (S94A), which encodes a putative enzyme involved in mycolic acid biosynthesis [48,87,88]. *Mycobacterium bovis* B2_7505, from a human patient, had both phenotypic resistance to INH as well as mutations in *kat*G (S315T) and *inh*A (S94A). Two isolates had the S315T mutation and according to Vilcheze et al., this variant is more often found in MDR_TB patients than in INH mono-resistant clinical isolates, hence it may be related to the higher transmission capabilities of these particular strains [50].

Rifampicin (RMP) is another important first-line drug for TB treatment and the most resistance to this drug arises from mutations in the *rpo*B gene, with the most frequent mutations seen at positions 516, 526, and 531 [89]. The most common mutation seen in our study was S450L and D435Y. The latter mutation (D435Y) is disputed on its association with rifampin resistance. However, its common occurrence in Ugandan isolates may be unique and represent a geographically restricted change because all isolates with the mutation were phenotypically resistant. The isolates screened in this study commonly exhibited the following *gyr*A mutations (E21Q, D668D), which, according to Lui et al. [83], were also seen in a fluoroquinolone susceptible strain, thus indicating that they may not be the source of fluoroquinolone resistance. Isolates B2_7505, Bz_31150, D_4155, and MR_4387 had a C206G nucleotide mutation in *gyr*A. Although fluoroqinolones (FQs) are proven to be very effective second-line anti-mycobacterial drugs for the treatment of drug-resistant TB, the increasing rate of drug resistance among them is a concern. 

The presence of these mutations (H57D, S315T, S94A) exclusively in the human *M. bovis* isolate suggests that these occurred after transmission from cattle. Genome analysis in this study identified *M. bovis* in humans and great apes, suggesting transmission from domesticated ruminants in the area due to a dynamic and changing interface, which has created an exposure opportunity. The acquisition, mutation, or loss of certain sections of genes could provide pathogens with peculiar advantages during infection and transmission and understanding these mechanisms could perhaps shed more light on the secrets these challenging organisms have that makes them so fastidious and successful. Mutations in key genes responsible for drug resistance have ensured that these organisms circumvent MTC therapy and perpetuate themselves in their hosts and in other susceptible hosts. MDR-TB and XDR-TB are a real threat to the achievements made in the fight against TB, one that needs a formidable arsenal of tools to overcome. 

### 4.5. Application of WGS/SNP Chip in the Field

Genome studies suggest that *M. bovis* transmission occurs between cattle, between cattle and humans, and potentially between humans [33,90,91,92] as well as other species, like wildlife [2,3,79,93]. Genotypic assays, like the Cepheid Xpert MTB/RIF (Cepheid, Sunnyvale, CA, USA) and Hain line-probe (Hain Lifescience, Nehren Germany) assays, provide a short turnaround time (less than a day) in the identification of mycobacteria; but they are not able to detect all resistance conferring mutations [94]. In our work, we used WGS to identify SNP differences in MTC species and determined those that had multi-drug resistance genes [81,94,95,96,97]. A total of 7250 SNPs was used to generate the phylogenetic tree that enabled us to determine the relatedness of the sequenced genomes to reference strains. This data should be regenerated with the addition of global MTC genome collections to help develop an affordable SNP chip to achieve sensitive, specific, and rapid results for MTC detection and typing to enable real-time monitoring of interspecies transmission.

## 5. Conclusions

Two of the 136 isolates were identified as *Mycobacterium bovis*. This likely reflects the study population at the JCRC. However, a number of the isolates were MDR-TB. The MTC species are strictly clonal and genetic material is only passed vertically from mother to daughter cells. This clonality facilitates phylogenetic analysis of the MTC complex, where variations occur exclusively by genomic mutations, like single nucleotide polymorphisms (SNPs), deletions, or insertions of small or large pieces of DNA [98,99]. In our work, we demonstrated the presence of *M. bovis* in a human and wildlife using archived samples from Uganda. This implies that more work in the pastoral communities would present a clearer picture of the TB disease dynamics and possibly the transmission patterns of MTCs between humans and animals. A subset of the SNPs we extracted from this dataset to create an SNP chip for field applications in order to detect MTCS quickly and accurately would be a useful field application. In addition, this work encourages a One Health approach between medical and veterinary departments in order to strengthen the health care and disease surveillance systems, and the need for translating science into policy [15,57,100]. 

## Figures and Tables

**Table 1:** Nucleotide sequence of primers used to determine the presence of *Mycobacterium bovis* [24,28].

Primer Type and Target Locus	Forward Primer	Reverse Primer	Size (bp)
16SrRNA	5′ ACG GTG GGT ACT AGG TGT GGG TTT C 3′	5′ TCT GCG ATT ACT AGC GAC TCC GAC TTC A 3′	543
IS6110	5′ TCA GCC GCG TCC ACG CCG CCA 3′	5′ CCG ACC GCT CCG ACC GAC GGT 3′	786
RD9 (Rv2073c)	5′ TCG CCG CTG CCA GAT GAG TC3′	5′ TTT GGG AGC CGC CGG TGG TGA TGA 3′	600
RD4 (Rv1510)	5′ GTG CGC TCC ACC CAA ATA GTT GC3′	5′ TGT CGA CCT GGG GCA CAA ATC AGT C 3′	1033
RD12 (Rv3120)	5′ GTC GGC GAT AGA CCA TGA GTC CGT CTC CAT3′	5′ GCG AAA AGT GGG CGG ATG CCA GAA TAG T 3′	404
RD1 (Rv3877/8)	5′ CGA CGG GTC TGA CGG CCA AAC TCA TC3′	5′ CTT GCT CGG TGG CCG GTT TTT CAG C 3′	999

**Table 2:** PCR results for 136 Pyranizamide (PZA )resistant *Mycobacterium tuberculosis* complex.

Regions of Difference (RD)	Suspected MTC	Frequency		Total Tested
		Positive +	Negative −	
16S rRNA	*Mycobacteria*	133	3	136
IS 6110	*Mycobacterium tuberculosis* complex	133	3	136
RD9	MTC other than *M. africanum* and *M. bovis*	126	7	133
RD4	*M. bovis*/*M. bovis* BCG/*M. caprae*	5	2	7
RD12	*M. bovis*/*M. bovis* BCG	0	2	2
RD1	*M. bovis*	2	0	2

**Table 3:** Drug resistance patterns.

Sample Type	Drug Resistance Patterns											
	NNNNN	NNNNR	NSSSR	RRRRR	RRRSR	RRSRR	RRSSR	SRRRR	SRRSR	SRSSR	SSSSR	Total
Blood								2				2
Bronchial wash											1	1
Gastric Aspirates											2	2
Isolate		2				1		2		2	12	19
Pleural fluid			1		1						1	3
Sputum	2			44	5	6	1	36	3	2	10	109
Total	2	2	1	44	6	7	1	40	3	4	26	136

S = sensitive, R = resistant N = not done. Order of first-line drugs and streptomycin: STM—streptomycin, INH—isoniazid, RMP—rifampicin, EMB—ethambutol, and PZA—pyrazinamide. NNNN—not done for all five TB drugs. NNNNR—not done for STM, INH, RMP, EMB, resistant to PZA. **NSSSR**—not done for STM; sensitive to INH, RMP, EMB, resistant to PZA. RRRRR—resistant to all five TB drugs. RRRSR—resistant to all drugs except EMB. RRSRR—resistant to all TB drugs except RMP, RRSSR—resistant to STM, INH, and PZA, sensitive to RMP, EMB. SRRRR—resistant to all drugs except STM. SRRSR—resistant to INH, RMP, PZA, sensitive to STM and EMB. SRSSR—resistant to RMP, PZA, sensitive to STM, RMP, and EMB. SSSSR—sensitive to all TB drugs except PZA.

**Table 4:** Mycobacterial genomes with Genbank accession numbers.

Bioproject ID	Biosample ID	Genome/Genbank Accession	Sample Name	Sample Source	Sample Type	Interval
PRJNA233395	SAMN02567763	JKAK00000000	*Mycobacterium tuberculosis* D 4155	Pleural biopsy	Human	Baseline
PRJNA233396	SAMN02567764	JKAJ00000000	*Mycobacterium tuberculosis* Kc 32216	Isolate	Human	Baseline
PRJNA233392	SAMN02567760	JKAN00000000	*Mycobacterium tuberculosis* Wt 21231	Sputum	Human	Baseline
PRJNA233393	SAMN02567761	JKAM00000000	*Mycobacterium bovis* Bz_ 31150	Bronchial wash	Chimpanzee	Unknown
PRJNA233394	SAMN02567762	JKAL00000000	*Mycobacterium bovis* B2_7505	Sputum	Human	Unknown
PRJNA233397	SAMN02567765	JKAI00000000	*Mycobacterium tuberculosis* Wt 21419	Sputum	Human	Baseline
PRJNA233398	SAMN02567766	JKAH00000000	*Mycobacterium bovis* Mr 4387	Sputum	Human	Unknown
PRJNA233399	SAMN02567767	JKAG00000000	*Mycobacterium tuberculosis* Kc 9614	Sputum	Human	3 mo. after start of treatment

**Table 5:** Global isolates used for phylogenetic analysis.

Isolate	Host	Country
Af2122_97 *M. bovis* reference strain	Cattle	United Kingdom
*M. bovis* B2_7505	Human	Uganda
*M. bovis* BZ_31150	Chimpanzee	Uganda
*M. tuberculosis WT 21231*	Human	Uganda
*M. tuberculosis WT 21419*	Human	Uganda
*M. tuberculosis KC_32216*	Human	Uganda
*M. tuberculosis KC 9614*	Human	Uganda
*M. tuberculosis D 4155*	Human	Uganda
*M. tuberculosis MR 438xz*	Human	Uganda
MBO_4303- *Mycobacterium bovis* strain 04-303	Wild boar living on free-range	Argentina
MBAN5v1 = *Mycobacterium bovis* Strain AN5	Used for Production of Purified Protein Derivative—Brazil	Brazil
ASM_93432v1 = *Mycobacterium bovis* strain SP38	Isolated from the lungs of a bovine	Sao Paolo, Brazil
NE_Elk	Elk, *Cervus canadensis*	Minnesota, USA
MBO_Consentino	Elk, *Cervus canadensis*	Minnesota, USA
MBO_Elk_94_0704-*Mycobacterium bovis*	Elk, *Cervus canadensis*	(USDA/APHIS/VS/NVSL)
*M. tuberculosis* KZN strain (Kwazulu Natal Isolate)	Human	South Africa
*M. tuberculosis* CDC 1551	Human	USA

**Table 6:** Drugs, genes, and drug-resistance associated mutations.

Drug	Gene	Gene Product	Mutations
INH	*Kat*G *inh*A	Catalase/peroxidase NADH-dependent enoyl-(ACP) reductase	S315T, R463L, A243P, V73A, W191G, Q525P S94A
RMP	*rpo*B	DNA directed RNA polymerase subunit beta	D435Y, D435V, S450L, M390T
PZA	*pnc*A	Pyrazinamidase/ nicotinamidase pncA	H57D, P54Q, A134V, V163A, V131F
STR	*rps*L *rrs*	rpsL 30S ribosomal protein S12 16S ribosomal RNA	None seenR309C, R338G, R173C
EMB	*emb*A *emb*C	Indolylacetylinositol arabinosyltransferase A Indolylacetylinositol arabinosyltransferase C	G884D, T608N T270I, V52X, M800V
OFX	gyrA	DNA gyrase subunit A	E21Q, S95T, G668D, D639A, R418W, D199G, T80A

**Table 7:** **Single nucleotide olymorphisms** (SNPs) identified in antibiotic resistance associated regions in *Mycobacterium tuberculosis* complex isolates.

Mutations in Target Gene (Corresponding Drugs)									
Isolate	Antimycobacterial Agent Resistance Profile	PncA- Rv2043c (PZA)	KatG—Rv1908c (INH)	RpoB- Rv0667 (RMP)	EmbA- Rv3794	EmbC Rv3793	InhA -Rv1484 (INH)	GyrA- Rv0006	16s rRNA-rrs Rvnr01
*M. bovis* Af2122/97	PZA	H57D	R463L	-	-	T270I	-	E21Q; S95T D639A; G668D	-
*M. tuberculosis* CDC_1551	n/a	-	-	-	-	V52X	-	E21Q; S95T G668D	-
*M. bovis* B2_7505	PZA, INH, RMP, EMB, STR	H57D	S315T R463L	D435Y	-	-	S94A	E21Q; S95T R418W; G668D	R309C
*M. bovis* Bz_31150	PZA	H57D	R463L	-	-	T270I	-	E21Q; S95T D199G; G668D	-
MBO Ravenel	n/a	-	R463L A243P	-	-	M800V	-	D639A G668D	-
MBO Elk	n/a	H57D	R463L	-	-	T270I	-	E21Q; S95T D639A; G668D	-
MBO Consentino	n/a	H57D	R463L V73A	-	-	T270I	-	E21Q; S95T D639A; G668D	-
Mtb MR 4387	STM, RMP, INH, EMB, PZA	A134V	R463L	D435V	G884D	-	-	E21Q; S95T G668D	R173C R338G
Mtb D4155	PZA	-	R463L	-	G884D	-	-	E21Q; S95T G668D	R338G
Mtb Kc 32216	PZA	_	-	-	-	-	-	E21Q; T80A S95T; G668D	-
Mtb Kc 9614	RMP, INH, EMB, PZA	Y(-4)C	S315T	S450L	T608N	-	-	E21Q; T80A S95T; G668D	-
WT 21419	RMP, INH, EMB, PZA	V163A	W191G	S450L	T608N	-	-	E21Q; T80A S95T; G668D	-
WT 21231	STM, RMP, INH, EMB, PZA	V131F	Q525P	M390T; S450L	-	-	-	E21Q	-

## Supplementary Materials

The following are available online at https://www.mdpi.com/2076-2607/7/8/221/s1, Figure S1: Studies from the Joint Clinical Research re (JCRC) studies from which pyrazinamide resistant samples were drawn; Figure S2: Distribution by age and gender of case patients with pyrazinamide (PZA) resistant isolates; Figure S3: Source of PZA samples tested for *Mycobacterium tuberculosis* complex; Figure S4: Resistance patterns for anti-tuberculosis drugs in PZA resistant isolates.

## References

[1] Cole S.T., Brosch R., Parkhill J., Garnier T., Churcher C., Harris D., Gordon S.V., Eiglmeier K., Gas S., Barry C.E. (1998). Deciphering the biology of Mycobacterium tuberculosis from the complete genome sequence. Nature.

[2] Banuls A.L., Sanou A., Anh N.T., Godreuil S. (2015). Mycobacterium tuberculosis: Ecology and evolution of a human bacterium. J. Med. Microbiol..

[3] Dos Vultos T., Mestre O., Rauzier J., Golec M., Rastogi N., Rasolofo V., Tonjum T., Sola C., Matic I., Gicquel B. (2008). Evolution and diversity of clonal bacteria: The paradigm of Mycobacterium tuberculosis. PLoS ONE.

[4] Gutierrez M.C., Brisse S., Brosch R., Fabre M., Omais B., Marmiesse M., Supply P., Vincent V. (2005). Ancient origin and gene mosaicism of the progenitor of Mycobacterium tuberculosis. PLoS Pathog..

[5] O’Reilly L.M., Daborn C.J. (1995). The epidemiology of Mycobacterium bovis infections in animals and man: A review. Tuber. Lung Dis..

[6] Michel A.L., Muller B., Van Helden P.D. (2010). Mycobacterium bovis at the animal-human interface: A problem, or not?. Vet. Microbiol..

[7] Nelson A.M. (1999). The cost of disease eradication. Smallpox and bovine tuberculosis. Ann. N. Y. Acad. Sci..

[8] Pesciaroli M., Alvarez J., Boniotti M.B., Cagiola M., Di Marco V., Marianelli C., Pacciarini M., Pasquali P. (2014). Tuberculosis in domestic animal species. Res. Vet. Sci..

[9] El-Sayed A., El-Shannat S., Kamel M., Castaneda-Vazquez M.A., Castaneda-Vazquez H. (2016). Molecular Epidemiology of Mycobacterium bovis in Humans and Cattle. Zoonoses. Public Health.

[10] Daborn C.J., Grange J.M., Kazwala R.R. (1996). The bovine tuberculosis cycle—An African perspective. J. Appl. Bacteriol..

[11] Cosivi O., Grange J.M., Daborn C.J., Raviglione M.C., Fujikura T., Cousins D., Robinson R.A., Huchzermeyer H.F.A.K., De Kantor I., Meslin F.X. (1998). Zoonotic tuberculosis due to Mycobacterium bovis in developing countries. Emerg. Infect. Dis..

[12] Berg S., Schelling E., Hailu E., Firdessa R., Gumi B., Erenso G., Gadisa E., Mengistu A., Habtamu M., Hussein J. (2015). Investigation of the high rates of extrapulmonary tuberculosis in Ethiopia reveals no single driving factor and minimal evidence for zoonotic transmission of Mycobacterium bovis infection. BMC Infect. Dis..

[13] Wedlock D.N., Skinner M.A., De Lisle G.W., Buddle B.M. (2002). Control of Mycobacterium bovis infections and the risk to human populations. Microbes. Infect..

[14] Muller B., Durr S., Alonso S., Hattendorf J., Laisse C.J., Parsons S.D., van Helden P.D., Zinsstag J. (2013). Zoonotic Mycobacterium bovis-induced tuberculosis in humans. Emerg. Infect. Dis..

[15] Kazwala R.R., Daborn C.J., Sharp J.M., Kambarage D.M., Jiwa S.F., Mbembati N.A. (2001). Isolation of Mycobacterium bovis from human cases of cervical adenitis in Tanzania: A cause for concern?. Int. J. Tuberc. Lung Dis..

[16] World Health Organization (2015). Tuberculosis.

[17] American Thoracic Society, Centers for Disease Control (1987). Mycobacterioses and the acquired immunodeficiency syndrome. Am. Rev. Respir. Dis..

[18] World Health Organization (2016). Uganda TB Profile.

[19] World Health Organization (2016). TB Diagnostics and Lab Strengthening.

[20] Oloya J., Muma J.B., Opuda-Asibo J., Djonne B., Kazwala R., Skjerve E. (2007). Risk factors for herd-level bovine-tuberculosis seropositivity in transhumant cattle in Uganda. Prev. Vet. Med..

[21] Oloya J., Opuda-Asibo J., Djonne B., Muma J.B., Matope G., Kazwala R., Skjerve E. (2006). Responses to tuberculin among Zebu cattle in the transhumance regions of Karamoja and Nakasongola district of Uganda. Trop. Anim. Health Prod..

[22] Oloya J., Kazwala R., Lund A., Opuda-Asibo J., Demelash B., Skjerve E., Johansen T.B., Djonne B. (2007). Characterisation of mycobacteria isolated from slaughter cattle in pastoral regions of Uganda. BMC Microbiol..

[23] Gallivan M., Shah N., Flood J. (2015). Epidemiology of human Mycobacterium bovis disease, California, USA, 2003–2011. Emerg. Infect. Dis..

[24] Huard R.C., Lazzarini L.C., Butler W.R., Van Soolingen D., Ho J.L. (2003). PCR-based method to differentiate the subspecies of the Mycobacterium tuberculosis complex on the basis of genomic deletions. J. Clin. Microbiol..

[25] Joint Clinical Research Centre 2016 Annual Report. https://www.jcrc.org.ug/resources/reports/annual-report-2016-sep-2017.

[26] Sambrook J., Fritsch E.F., Maniatis T. (1989). Molecular Cloning.

[27] Sambrook J., Russell D.W. (2006). Storage of bacterial cultures growing on solid medium. CSH Protoc..

[28] Huard R.C., Fabre M., De Haas P., Lazzarini L.C., Van Soolingen D., Cousins D., Ho J.L. (2006). Novel genetic polymorphisms that further delineate the phylogeny of the Mycobacterium tuberculosis complex. J. Bacteriol..

[29] Pym A.S., Brodin P., Brosch R., Huerre M., Cole S.T. (2002). Loss of RD1 contributed to the attenuation of the live tuberculosis vaccines Mycobacterium bovis BCG and Mycobacterium microti. Mol. Microbiol..

[30] Thorne N., Borrell S., Evans J., Magee J., Garcia De Viedma D., Bishop C., Gonzalez-Martin J., Gharbia S., Arnold C. (2011). IS6110-based global phylogeny of Mycobacterium tuberculosis. Infect. Genet. Evol..

[31] Brosch R., Gordon S.V., Marmiesse M., Brodin P., Buchrieser C., Eiglmeier K., Garnier T., Gutierrez C., Hewinson G., Kremer K. (2002). A new evolutionary scenario for the Mycobacterium tuberculosis complex. Proc. Natl. Acad. Sci. USA.

[32] Kamerbeek J., Schouls L., Kolk A., Van Agterveld M., Van Soolingen D., Kuijper S., Bunschoten A., Molhuizen H., Shaw R., Goyal M. (1997). Simultaneous detection and strain differentiation of Mycobacterium tuberculosis for diagnosis and epidemiology. J. Clin. Microbiol..

[33] Van Soolingen D., Hermans P.W., De Haas P.E., Soll D.R., Van Embden J.D. (1991). Occurrence and stability of insertion sequences in Mycobacterium tuberculosis complex strains: Evaluation of an insertion sequence-dependent DNA polymorphism as a tool in the epidemiology of tuberculosis. J. Clin. Microbiol..

[34] Asiimwe B.B., Asiimwe J., Kallenius G., Ashaba F.K., Ghebremichael S., Joloba M., Koivula T. (2009). Molecular characterisation of Mycobacterium bovis isolates from cattle carcases at a city slaughterhouse in Uganda. Vet. Rec..

[35] Tuberculosis (2016). Broad Institute. https://www.broadinstitute.org/infectious-disease-and-microbiome/tuberculosis.

[36] Olive D. (2014). TB-ARC M.Bovis.1..

[37] Darling A.C., Mau B., Blattner F.R., Perna N.T. (2004). Mauve: Multiple alignment of conserved genomic sequence with rearrangements. Genome. Res..

[38] Darling A.E., Mau B., Perna N.T. (2010). Progressivemauve: Multiple genome alignment with gene gain, loss and rearrangement. PLoS ONE.

[39] Tamura K., Stecher G., Peterson D., Filipski A., Kumar S. (2013). MEGA6: Molecular Evolutionary Genetics Analysis version 6.0. Mol. Biol. Evol..

[40] Aziz R.K., Bartels D., Best A.A., DeJongh M., Disz T., Edwards R.A., Formsma K., Gerdes S., Glass E.M., Kubal M. (2008). The RAST Server: Rapid annotations using subsystems technology. BMC Genom..

[41] National Center for Biotechnology Information (2016). Basic Local Alignment Search Tool.

[42] National Center for Biotechnology Information (2016). TB-ARC-M. Bovis.

[43] National Center for Biotechnology Information (2016). Bioproject.

[44] Wanzala S.I., Nakavuma J., Travis D.A., Kia P., Ogwang S., Sreevatsan S. (2015). Draft Genome Sequences of Mycobacterium bovis BZ 31150 and Mycobacterium bovis B2 7505, Pathogenic Bacteria Isolated from Archived Captive Animal Bronchial Washes and Human Sputum Samples in Uganda. Genome. Announc..

[45] Sreevatsan S., Pan X., Stockbauer K.E., Connell N.D., Kreiswirth B.N., Whittam T.S., Musser J.M. (1997). Restricted structural gene polymorphism in the Mycobacterium tuberculosis complex indicates evolutionarily recent global dissemination. Proc. Natl. Acad. Sci. USA.

[46] Sreevatsan S., Pan X., Zhang Y., Kreiswirth B.N., Musser J.M. (1997). Mutations associated with pyrazinamide resistance in pncA of Mycobacterium tuberculosis complex organisms. Antimicrob. Agents Chemother..

[47] Sreevatsan S., Escalante P., Pan X., Gillies D.A., Siddiqui S., Khalaf C.N., Kreiswirth B.N., Bifani P., Adams L.G., Ficht T. (1996). Identification of a polymorphic nucleotide in oxyR specific for Mycobacterium bovis. J. Clin. Microbiol..

[48] Seifert M., Catanzaro D., Catanzaro A., Rodwell T.C. (2015). Genetic mutations associated with isoniazid resistance in Mycobacterium tuberculosis: A systematic review. PLoS ONE.

[49] Brammacharry U., Muthaiah M. (2014). Characterization of rpsL Gene Mutations in Streptomycin-Resistant Mycobacterium tuberculosis Isolates. Am. J. Microbiol. Res..

[50] Vilcheze C., Jacobs W.R. (2014). Resistance to Isoniazid and Ethionamide in Mycobacterium tuberculosis: Genes, Mutations, and Causalities. Microbiol. Spectr..

[51] Gagneux S. (2012). Host-pathogen coevolution in human tuberculosis. Philos. Trans. R. Soc. B Biol. Sci..

[52] Sekiguchi J., Miyoshi-Akiyama T., Augustynowicz-Kopec E., Zwolska Z., Kirikae F., Toyota E., Kobayashi I., Morita K., Kudo K., Kato S. (2007). Detection of multidrug resistance in Mycobacterium tuberculosis. J. Clin. Microbiol..

[53] Dominguez J., Boettger E.C., Cirillo D., Cobelens F., Eisenach K.D., Gagneux S., Hillemann D., Horsburgh R., Molina-Moya B., Niemann S. (2016). Clinical implications of molecular drug resistance testing for Mycobacterium tuberculosis: A TBNET/RESIST-TB consensus statement. Int. J. Tuberc. Lung Dis..

[54] Avalos E., Catanzaro D., Catanzaro A., Ganiats T., Brodine S., Alcaraz J., Rodwell T. (2015). Frequency and geographic distribution of gyrA and gyrB mutations associated with fluoroquinolone resistance in clinical Mycobacterium tuberculosis isolates: A systematic review. PLoS ONE.

[55] Malik S., Willby M., Sikes D., Tsodikov O.V., Posey J.E. (2012). New insights into fluoroquinolone resistance in Mycobacterium tuberculosis: Functional genetic analysis of gyrA and gyrB mutations. PLoS ONE.

[56] Mayer C., Takiff H. (2014). The Molecular Genetics of Fluoroquinolone Resistance in Mycobacterium tuberculosis. Microbiol. Spectr..

[57] Kankya C., Muwonge A., Olet S., Munyeme M., Biffa D., Opuda-Asibo J., Skjerve E., Oloya J. (2010). Factors associated with pastoral community knowledge and occurrence of mycobacterial infections in human-animal interface areas of Nakasongola and Mubende districts, Uganda. BMC Public Health.

[58] Cousins D.V. (2001). Mycobacterium bovis infection and control in domestic livestock. Rev. Sci. Technol..

[59] Cleaveland S., Shaw D.J., Mfinanga S.G., Shirima G., Kazwala R.R., Eblate E., Sharp M. (2007). Mycobacterium bovis in rural Tanzania: Risk factors for infection in human and cattle populations. Tuberculosis.

[60] Cadmus S., Palmer S., Okker M., Dale J., Gover K., Smith N., Jahans K., Hewinson R.G., Gordon S.V. (2006). Molecular analysis of human and bovine tubercle bacilli from a local setting in Nigeria. J. Clin. Microbiol..

[61] Brudey K., Driscoll J.R., Rigouts L., Prodinger W.M., Gori A., Al-Hajoj S.A., Allix C., Aristimuno L., Arora J., Baumanis V. (2006). Mycobacterium tuberculosis complex genetic diversity: Mining the fourth international spoligotyping database (SpolDB4) for classification, population genetics and epidemiology. BMC Microbiol..

[62] Ayele W.Y., Neill S.D., Zinsstag J., Weiss M.G., Pavlik I. (2004). Bovine tuberculosis: An old disease but a new threat to Africa. Int. J. Tuberc. Lung Dis..

[63] Etter E., Donado P., Jori F., Caron A., Goutard F., Roger F. (2006). Risk analysis and bovine tuberculosis, a re-emerging zoonosis. Ann. N. Y. Acad. Sci..

[64] Alemayehu R., Girmay M., Gobena A. (2008). Bovine tuberculosis is more prevalent in cattle owned by farmers with active tuberculosis in central Ethiopia. Vet. J..

[65] Aylate A., Shah S.N., Aleme H., Gizaw T.T. (2013). Bovine tuberculosis: Prevalence and diagnostic efficacy of routine meat inspection procedure in Woldiya municipality abattoir north Wollo zone, Ethiopia. Trop. Anim. Health Prod..

[66] Ameni G., Vordermeier M., Firdessa R., Aseffa A., Hewinson G., Gordon S.V., Berg S. (2011). Mycobacterium tuberculosis infection in grazing cattle in central Ethiopia. Vet. J..

[67] Ameni G., Tadesse K., Hailu E., Deresse Y., Medhin G., Aseffa A., Hewinson G., Vordermeier M., Berg S. (2013). Transmission of Mycobacterium tuberculosis between farmers and cattle in central Ethiopia. PLoS ONE.

[68] Ameni G., Erkihun A. (2007). Bovine tuberculosis on small-scale dairy farms in Adama Town, central Ethiopia, and farmer awareness of the disease. Rev. Sci. Technol..

[69] Ameni G., Aseffa A., Engers H., Young D., Hewinson G., Vordermeier M. (2006). Cattle husbandry in Ethiopia is a predominant factor affecting the pathology of bovine tuberculosis and gamma interferon responses to mycobacterial antigens. Clin. Vaccine Immunol..

[70] Roug A., Perez A., Mazet J.A., Clifford D.L., VanWormer E., Paul G., Kazwala R.R., Smith W.A. (2014). Comparison of intervention methods for reducing human exposure to Mycobacterium bovis through milk in pastoralist households of Tanzania. Prev. Vet. Med..

[71] Berg S., Firdessa R., Habtamu M., Gadisa E., Mengistu A., Yamuah L., Ameni G., Vordermeier M., Robertson B.D., Smith N.H. (2009). The burden of mycobacterial disease in ethiopian cattle: Implications for public health. PLoS ONE.

[72] Ramirez-Busby S.M., Valafar F. (2015). Systematic review of mutations in pyrazinamidase associated with pyrazinamide resistance in Mycobacterium tuberculosis clinical isolates. Antimicrob. Agents Chemother..

[73] Cirillo D.M., Cabibbe A.M., De Filippo M.R., Trovato A., Simonetti T., Rossolini G.M., Tortoli E. (2016). Use of WGS in Mycobacterium tuberculosis routine diagnosis. Int. J. Mycobacteriol..

[74] Wattam A.R., Abraham D., Dalay O., Disz T.L., Driscoll T., Gabbard J.L., Gillespie J.J., Gough R., Hix D., Kenyon R. (2014). PATRIC, the bacterial bioinformatics database and analysis resource. Nucleic Acids Res..

[75] Le Chevalier F., Cascioferro A., Majlessi L., Herrmann J.L., Brosch R. (2014). Mycobacterium tuberculosis evolutionary pathogenesis and its putative impact on drug development. Future Microbiol..

[76] Moran N.A. (2002). Microbial minimalism: Genome reduction in bacterial pathogens. Cell.

[77] Votintseva A.A., Bradley P., Pankhurst L., Del Ojo Elias C., Loose M., Nilgiriwala K., Chatterjee A., Smith E.G., Sanderson N., Walker T.M. (2017). Same-day diagnostic and surveillance data for tuberculosis via whole genome sequencing of direct respiratory samples. J. Clin. Microbiol..

[78] Joshi D., Harris N.B., Waters R., Thacker T., Mathema B., Krieswirth B., Sreevatsan S. (2012). Single nucleotide polymorphisms in the Mycobacterium bovis genome resolve phylogenetic relationships. J. Clin. Microbiol..

[79] Garcia-Betancur J.C., Menendez M.C., Del Portillo P., Garcia M.J. (2012). Alignment of multiple complete genomes suggests that gene rearrangements may contribute towards the speciation of Mycobacteria. Infect. Genet. Evol..

[80] Salipante S.J., SenGupta D.J., Cummings L.A., Land T.A., Hoogestraat D.R., Cookson B.T. (2015). Application of whole-genome sequencing for bacterial strain typing in molecular epidemiology. J. Clin. Microbiol..

[81] Takiff H.E., Feo O. (2015). Clinical value of whole-genome sequencing of Mycobacterium tuberculosis. Lancet Infect. Dis..

[82] Jagielski T., Minias A., Van Ingen J., Rastogi N., Brzostek A., Zaczek A., Dziadek J. (2016). Methodological and Clinical Aspects of the Molecular Epidemiology of Mycobacterium tuberculosis and Other Mycobacteria. Clin. Microbiol. Rev..

[83] Liu F., Hu Y., Wang Q., Li H.M., Gao G.F., Liu C.H., Zhu B. (2014). Comparative genomic analysis of Mycobacterium tuberculosis clinical isolates. BMC Genom..

[84] Whitfield M.G., Soeters H.M., Warren R.M., York T., Sampson S.L., Streicher E.M., Van Helden P.D., Van Rie A. (2015). A Global Perspective on Pyrazinamide Resistance: Systematic Review and Meta-Analysis. PLoS ONE.

[85] Scorpio A., Zhang Y. (1996). Mutations in pncA, a gene encoding pyrazinamidase/nicotinamidase, cause resistance to the antituberculous drug pyrazinamide in tubercle bacillus. Nat. Med..

[86] Maslov D.A., Zaichikova M.V., Chernousova L.N., Shur K.V., Bekker O.B., Smirnova T.G., Larionova E.E., Andreevskaya S.N., Zhang Y., Danilenko V.N. (2015). Resistance to pyrazinamide in Russian Mycobacterium tuberculosis isolates: pncA sequencing versus Bactec MGIT 960. Tuberculosis.

[87] Vilcheze C., Wang F., Arai M., Hazbon M.H., Colangeli R., Kremer L., Weisbrod T.R., Alland D., Sacchettini J.C., Jacobs W.R. (2006). Transfer of a point mutation in Mycobacterium tuberculosis inhA resolves the target of isoniazid. Nat. Med..

[88] Aye K.S., Nakajima C., Yamaguchi T., Win M.M., Shwe M.M., Win A.A., Lwin T., Nyunt W.W., Ti T., Suzuki Y. (2016). Genotypic characterization of multi-drug-resistant Mycobacterium tuberculosis isolates in Myanmar. J. Infect. Chemother..

[89] Khosravi A.D., Goodarzi H., Alavi S.M. (2012). Detection of genomic mutations in katG, inhA and rpoB genes of Mycobacterium tuberculosis isolates using polymerase chain reaction and multiplex allele-specific polymerase chain reaction. Braz. J. Infect. Dis..

[90] Vordermeier H.M., Perez De Val B., Buddle B.M., Villarreal-Ramos B., Jones G.J., Hewinson R.G., Domingo M. (2014). Vaccination of domestic animals against tuberculosis: Review of progress and contributions to the field of the TBSTEP project. Res. Vet. Sci..

[91] Tschopp R., Aseffa A., Schelling E., Berg S., Hailu E., Gadisa E., Habtamu M., Argaw K., Zinsstag J. (2010). Bovine tuberculosis at the wildlife-livestock-human interface in Hamer Woreda, South Omo, Southern Ethiopia. PLoS ONE.

[92] Srivastava K., Chauhan D.S., Gupta P., Singh H.B., Sharma V.D., Yadav V.S., Sreekumaran, Thakral S.S., Dharamdheeran J.S., Nigam P. (2008). Isolation of Mycobacterium bovis and M-tuberculosis from cattle of some farms in north India—Possible relevance in human health. Indian J. Med. Res..

[93] Ehrt S., Rhee K., Schnappinger D. (2015). Mycobacterial genes essential for the pathogen’s survival in the host. Immunol. Rev..

[94] Pankhurst L.J., Del Ojo Elias C., Votintseva A.A., Walker T.M., Cole K., Davies J., Fermont J.M., Gascoyne-Binzi D.M., Kohl T.A., Kong C. (2016). Rapid, comprehensive, and affordable mycobacterial diagnosis with whole-genome sequencing: A prospective study. Lancet Respir. Med..

[95] Sabat A.J., Budimir A., Nashev D., Sa-Leao R., Van Dijl J., Laurent F., Grundmann H., Friedrich A.W., ESGEM (2013). Overview of molecular typing methods for outbreak detection and epidemiological surveillance. Eurosurveillance.

[96] Kjeldsen M.K., Bek D., Rasmussen E.M., Prieme A., Thomsen V.O. (2009). Line probe assay for differentiation within Mycobacterium tuberculosis complex. Evaluation on clinical specimens and isolates including Mycobacterium pinnipedii. Scand. J. Infect. Dis..

[97] Harris S.R., Okoro C.K. (2014). Whole-Genome Sequencing for Rapid and Accurate Identification of Bacterial Transmission Pathways. New Approaches Prokaryotic Syst..

[98] Zakham F., Aouane O., Ussery D., Benjouad A., Ennaji M.M. (2012). Computational genomics-proteomics and Phylogeny analysis of twenty one mycobacterial genomes (Tuberculosis and non Tuberculosis strains). Microb. Inf. Exp..

[99] Schurch A.C., van Soolingen D. (2012). DNA fingerprinting of Mycobacterium tuberculosis: From phage typing to whole-genome sequencing. Infect. Genet. Evol..

[100] Hasegawa M., Kishino H., Yano T. (1985). Dating of the human-ape splitting by a molecular clock of mitochondrial DNA. J. Mol. Evol..

