# Supplementary figures and images for: Retrospective Analysis of Archived Pyrazinamide Resistant Mycobacterium tuberculosis Complex Isolates from Uganda—Evidence of Interspecies Transmission
Source: Microorganisms. 2019 Jul 29;7(8):221. doi: 10.3390/microorganisms7080221 (PMC6723201; doi:10.3390/microorganisms7080221)

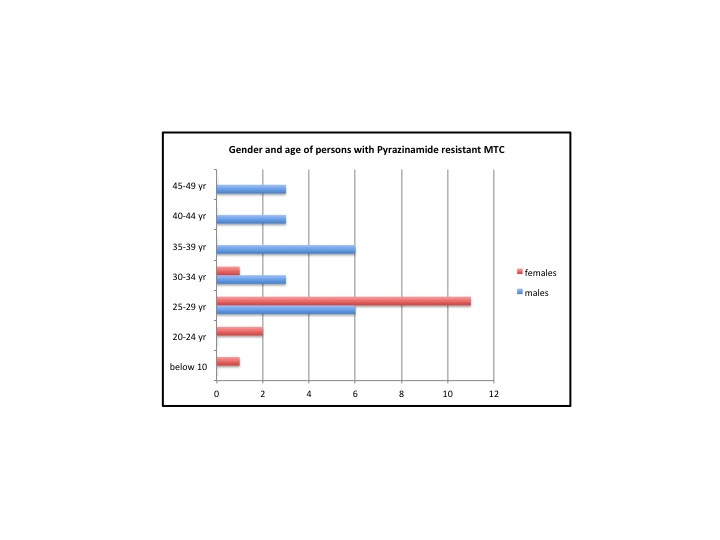

Supplement: Supplementary file 1 [file microorganisms-07-00221-s001.zip › Supp_Figure S2.jpg]

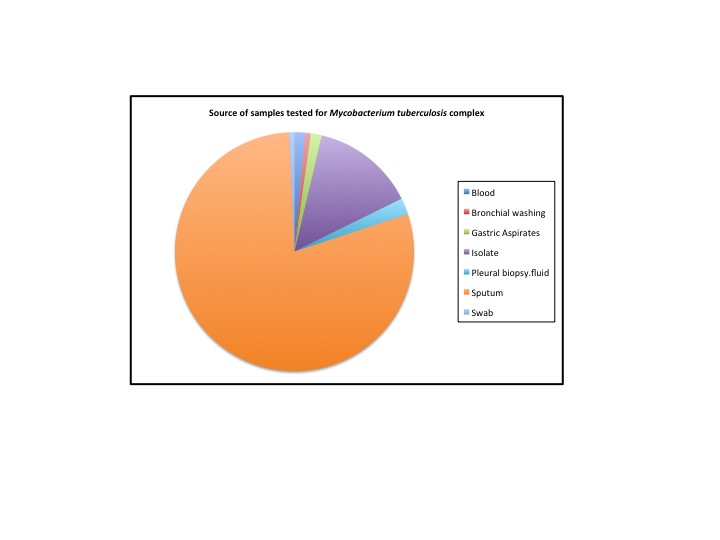

Supplement: Supplementary file 1 [file microorganisms-07-00221-s001.zip › Supp_Figure S3.jpg]

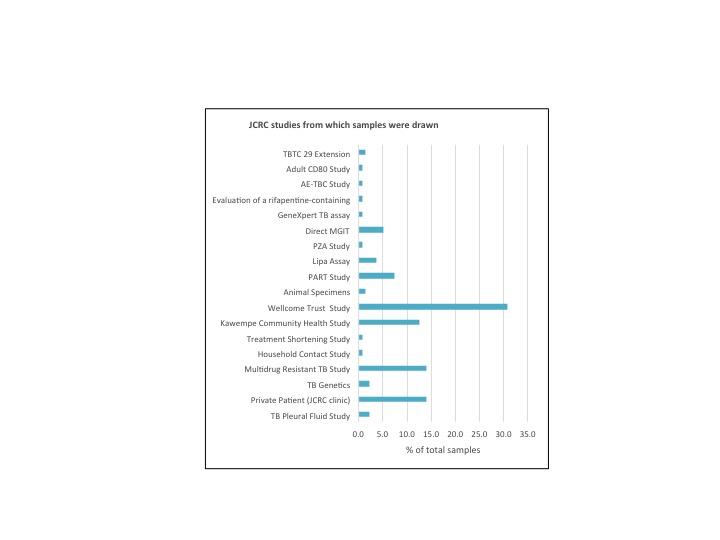

Supplement: Supplementary file 1 [file microorganisms-07-00221-s001.zip › Supp_Figure S1.jpg]

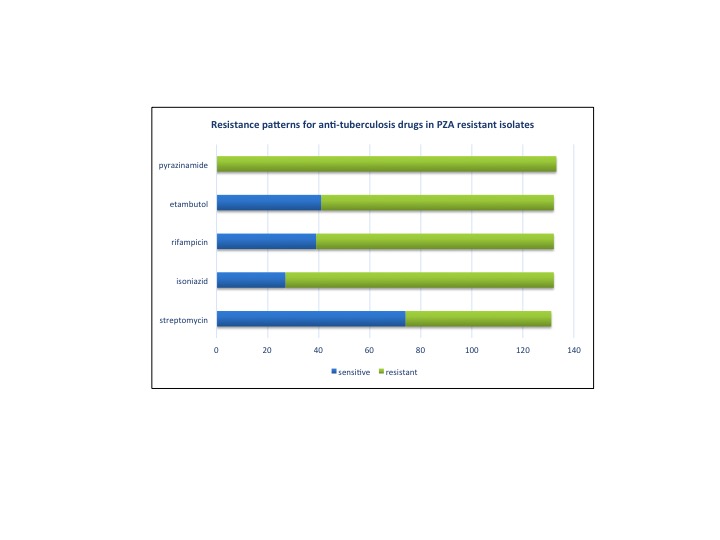

Supplement: Supplementary file 1 [file microorganisms-07-00221-s001.zip › Supp_Figure S4.jpg]
